# Radiological findings on computed tomography examinations of adult patients in the emergency department of a tertiary care hospital

**DOI:** 10.1590/0100-3984.2024.0068-en

**Published:** 2025-01-06

**Authors:** Ana Júlia Xavier da Cruz Soares, Bianca Tessele, André Luiz Picoloto, Luciana Estacia Ambros

**Affiliations:** 1 Escola de Medicina - Universidade de Passo Fundo (UPF), Passo Fundo, RS, Brazil

**Keywords:** Tomography, X-ray computed, Emergencies, Diagnostic imaging, Tomografia computadorizada, Emergências, Diagnóstico por imagem

## Abstract

**Objective:**

To identify and analyze the main findings on computed tomography (CT) scans ordered in the emergency department of a tertiary care hospital.

**Materials and Methods:**

This was a cross-sectional observational study conducted through analysis of CT scans of the head, chest, and abdomen of all patients admitted to the emergency department of a tertiary care hospital over a period of four months.

**Results:**

Among a sample of 331 patients, pathological radiological findings were observed in 59.2%, with the highest number of alterations being observed on abdominal CT, followed by chest CT. The most prevalent findings were as follows: in the abdomen-obstructive uropathy (in 12.2%), pyelonephritis (in 6.4%), cholecystitis (in 5.8%), and enterocolitis (in 5.8%); in the chest-pneumonia (in 23.7%), pleural effusion (in 12.9%), and pulmonary contusion (in 5.4%); and in the head-extracranial soft tissue edema (in 11.9%), stroke (in 10.7%), and brain contusion (in 7.9%).

**Conclusion:**

Our findings demonstrate the importance of using CT for proper diagnosis and clinical management in emergency departments. Our data also contribute to establishing the most prevalent radiological findings in each anatomical region, thus promoting rapid, efficient clinical practice in emergency settings.

## Abstract (pt)

**Objetivo:**

Identificar e analisar os principais achados tomográficos solicitados pelo setor de emergência de um hospital terciário.

**Materiais e Métodos:**

Estudo observacional transversal realizado por meio da análise de tomografia computadorizada (TC) de crânio, tórax e abdome de todos os pacientes admitidos no setor de emergência de um hospital de alta complexidade em um período de quatro meses.

**Resultados:**

Com base na amostra total de 331 pacientes, observaram-se achados radiológicos patológicos em 59,2% dos pacientes, sendo as TCs de abdome com maior número de alterações, seguidas de TCs de tórax. Os achados patológicos mais prevalentes no abdome foram uropatia obstrutiva (12,2%), pielonefrite (6,4%), colecistite (5,8%) e enterocolite (5,8%); no tórax foram pneumonia (23,7%), derrame pleural (12,9%) e contusão pulmonar (5,4%); e no crânio foram aumento de partes moles extracranianas (11,9%), acidente vascular cerebral (10,7%) e contusão encefálica (7,9%).

**Conclusão:**

Demonstrou-se a importância do uso de exames tomográficos em pacientes do setor de emergência para adequado diagnóstico e manejo clínico. Além disso, os dados deste trabalho contribuem para estabelecer quais os achados radiológicos mais prevalentes de acordo com cada área anatômica, auxiliando, assim, a prática clínica rápida e eficiente em ambientes de emergência.

## INTRODUCTION

Advances in medical imaging technology, particularly in computed tomography (CT), have revolutionized the practice of emergency medicine, allowing rapid, accurate assessment of patients with acute conditions. At tertiary care hospitals, where complex and high-severity cases are concentrated, the analysis of radiological findings from CT examinations in the emergency department plays a crucial role in triage, diagnosis, and therapeutic management. However, despite the importance of this radiological examination, there is a significant gap in the scientific literature regarding the most prevalent CT findings in adult patients presenting to the emergency department.

The lack of specific data on the most common radiological findings in these situations can pose a challenge for emergency physicians, who often need to make quick, accurate decisions based on the information provided by imaging examinations. Knowing which findings are most prevalent in different acute clinical conditions is essential to guide the initial practice of physicians, allowing a more targeted, efficient approach to patient care.

This article proposes an evaluation of the epidemiological profile of the most common radiological findings from CT examinations performed in the emergency department of a tertiary care hospital in Brazil. The identification and understanding of these findings have the potential to improve clinical practice, optimizing the triage, diagnosis, and therapeutic management in emergency situations, resulting in better outcomes for patients treated in this critical setting.

## MATERIALS AND METHODS

This was a prospective cross-sectional study of the findings from CT examinations of 331 patients admitted to the emergency department of a tertiary care hospital between September 1 and December 15 of 2023. The study was approved by the research ethics committee of the hospital (Reference nos. 6,416,574 and 72964223.7.0000.5342). All participating patients gave written informed consent.

The inclusion criteria were being ≥ 18 years of age, having been admitted to the emergency department of a tertiary care hospital, and having undergone CT examination of the head, chest, or abdomen, with and without contrast. Patients who were unconscious were excluded, as were those who were hemodynamically unstable, those previously diagnosed with a neoplasm or a chronic disease, and those for whom the examinations were of insufficient technical quality to allow an adequate analysis of the images.

Patient demographics, including age, sex, and chief complaint at presentation to the emergency department, were obtained by reviewing the hospital medical records. In addition, clinical indications for abdominal, chest, and head CT scans were recorded, as was whether the emergencies were traumatic or non-traumatic.

All CT scans were ordered by emergency physicians at the tertiary hospital and performed in accordance with the standard protocols for each relevant anatomical region, including images acquired with and without contrast.

The CT examinations were performed in a 64-slice scanner (Somatom Sensation 64; Siemens, Erlangen, Germany) or a 16-slice scanner (Optima CT520; GE HealthCare, Milwaukee, WI, USA). The resulting images were analyzed and selected by a radiologist with over seven years of experience, using a viewer for digital images (Arya/PACS Aurora, version 24.11.0; Pixeon, São Caetano do Sul, Brazil). From the selected images, the relevant radiological findings in each anatomical region were identified and recorded, including acute pathologies, traumatic injuries, structural alterations, and other conditions relevant to clinical practice in the emergency department.

### Statistical analysis

Continuous variables are expressed as means and standard deviations or as medians and interquartile ranges. Categorical variables are expressed as counts and percentages. No imputation was performed for missing data. All analyses were performed with Microsoft Excel and with the IBM SPSS Statistics software package, version 22.0 (IBM Corp., Armonk, NY, USA).

## RESULTS

The study sample consisted of 331 patients, of whom 174 (52.6%) were women and 157 (47.4%) were men. The mean age was 53.5 ± 21.0 years (range, 30-75 years). Our data differ from those of other studies in that patients under 18 years of age, unconscious patients, and hemodynamically unstable patients were excluded. Of the 331 patients in the sample, 263 (79.5%) underwent CT of only one anatomical segment, 35 (10.6%) underwent CT of two anatomical segments, and 33 (10.0%) underwent CT of three anatomical segments, resulting in a total of 432 CT scans. Of those, 23 were excluded because of inadequate image quality. Therefore, the final sample comprised 409 CT scans of 331 patients.

Traumatic emergencies accounted for 107 (32.3%) of the cases included in the study, and non-traumatic emergencies accounted for 224 (67.7%). There were 183 CT examinations of the head-88 (48.1%) for traumatic emergencies and 95 (51.9%) for non-traumatic emergencies-99 CT examinations of the chest-45 (45.5%) for traumatic emergencies and 54 (54.4%) for non-traumatic emergencies-and 150 CT examinations of the abdomen-43 (28.7%) for traumatic emergencies and 107 (71.3%) for non-traumatic emergencies. The CT examinations were performed with contrast in 185 patients (55.9%) and without contrast in 146 (44.1%). Incidental findings suggestive of neoplasia were reported for 15 patients (4.5%).

Of the 331 patients evaluated, 196 (59.2%) presented positive CT findings for acute alterations. Of the 183 CT scans of the head, 177 were analyzed and 72 (40.6%) of those presented acute pathological findings. Of the 99 CT scans of the chest, 93 were analyzed and 49 (52.6%) of those presented acute pathological findings. Of 150 CT scans of the abdomen, 139 were analyzed and 79 (56.8%) of those presented acute pathological findings. Of the 72 instances in which a head CT revealed acute pathological findings, 45 (62.5%) were classified as traumatic, as were 13 (26.5%) of the 49 instances in which a chest CT revealed such findings and five (6.3%) of the 79 instances in which an abdominal CT revealed such findings.

Among the 72 head CT scans with acute pathological findings, the main findings were increased extracranial soft tissue (subgaleal hematoma; in 11.9%), stroke (in 10.7%), brain contusion (in 7.9%), and intracranial hemorrhage (in 5.1%). Other head CT findings are described in Table 1. Among the 49 chest CT scans with acute pathological findings, the main alterations found were pneumonia (in 23.7%), pleural effusion (in 12.9%), pulmonary contusion (in 5.4%), and bone fracture (in 4.3%). Other chest CT findings are described in Table 2. Among the 79 abdominal CT scans with acute pathological findings, the main alterations found were obstructive uropathy (in 12.2%), pyelonephritis (in 6.4%), cholecystitis (in 5.8%), enterocolitis (in 5.8%), and diverticulitis (in 5.0%). Other abdominal CT findings are described in Table 3.

**Table 1:** Radiological findings on CT scans of the head, by the type of emergency (traumatic and non-traumatic).

CT finding	Total (N = 72) n (%)	Traumatic (n = 45) n (%)	Non-traumatic (n = 27) n (%)
Extracranial soft tissue			
edema	21 (11.9)	21 (100)	-
Stroke	19 (10.7)	1 (5.2)	18 (94.7)
Brain contusion	14 (7.9)	14 (100)	-
Intracranial hemorrhage	9 (5.1)	3 (33.3)	6 (66.6)
Subdural hematoma	2 (1.1)	2 (100)	-
Subarachnoid hemorrhage	2 (1.1)	1(50)	1(50)
Brain mass	2 (1.1)	-	2 (100)
Diffuse axonal injury	2 (1.1)	2 (100)	-
Epidural hematoma	1 (0.6)	1 (100)	-

**Table 2:** Radiological findings on CT scans of the chest, by the type of emergency (traumatic and non-traumatic).

CT finding	Total (N = 49) n (%)	Traumatic (n = 13) n (%)	Non-traumatic (n = 36) n (%)
Pneumonia	22 (23.7)	1 (4.5)	21 (95.4)
Pleural effusion	12 (12.9)	3(25)	9(75)
Pulmonary contusion	5 (5.4)	4(80)	1(20)
Bone fracture	4 (4.3)	4(100)	-
Pulmonary congestion	2 (2.2)	-	2(100)
Aortic dissection	1 (0.3)	-	1(100)
Pneumothorax	1 (0.3)	1(100)	-
Cardiomyopathy	1 (0.3)	-	1(100)
Tuberculosis	1 (0.3)	-	1(100)

**Table 3:** Radiological findings on CT scans of the abdomen, by the type of emergency (traumatic and non-traumatic).

CT finding	Total (N = 79) n (%)	Traumatic (n = 5) n (%)	Non-traumatic (n = 74) n (%)
Obstructive uropathy	17 (12,2)	-	17(100)
Pyelonephritis	9 (6,4)	-	9 (100)
Cholecystitis	8 (5,8)	1 (12,5)	7 (87,5)
Enterocolitis	8 (5,8)	-	8 (100)
Diverticulitis	7(5)	-	7 (100)
Intestinal subocclusion	6 (4,3)	-	6 (100)
Bowel obstruction	5 (3,6)	-	5 (100)
Appendicitis	5 (3,6)	-	5 (100)
Nephrolithiasis	5 (3,6)	-	5 (100)
Visceral perforation	4 (2,9)	4(100)	-
Pancreatitis	3 (2,2)	-	3 (100)
Aortic dissection	1 (0,7)	-	1 (100)
Abdominal aortic aneurysm	1 (0,7)	-	1 (100)

## DISCUSSION

Our finding that 52.6% of the adult patients presenting for emergency treatment at our tertiary care hospital during the study period were women is in keeping with the findings of Lin et al.^(1)^, who reported a female prevalence of 57.6% among such patients. This sex-related discrepancy in treatment seeking behavior might be attributable to the male culture of greater exposure to risks and fewer health concerns^(2)^.

Our study sample was composed of adults between 30 and 75 years of age, with a mean age of 53.5 years. It should be borne in mind that we excluded pediatric patients (those < 18 years of age) from the sample. In our sample, the vast majority (79.5%) of CT scans were ordered for only one of the three anatomical regions under investigation, whereas 10.6% were ordered for two of the regions and 10.0% were ordered for all three regions (head, chest, and abdomen).

In emergency settings worldwide, there is a tendency to excessively order excessive numbers of ancillary imaging examinations, few of which produce relevant findings, as demonstrated in the study conducted by Seidel et al.^(3)^, in which 56% of the examinations were “without acute alteration”. However, despite the similarities between the studies (both of CT scans ordered in the emergency department of a tertiary care hospital, with cases subdivided into traumatic and non-traumatic emergencies), our study sample differed from that evaluated by those authors, given that 59.2% of our patients presented acute alterations on the CT scans.

In our sample, 32.3% of the patients underwent CT examinations due to traumatic emergencies. However, there were discrepancies among the anatomical regions in relation to the presence of acute radiological findings in those cases, with trauma being the cause of the emergency in 62.5% of the positive head CTs, 26.5% of the chest CTs, and only 6.3% of the abdominal CTs. The group of patients with traumatic emergencies was composed mainly of polytrauma victims of car accidents, and we can therefore suggest that there is an association between traumatic emergencies and the request for CT in multiple regions, which would explain the propensity to order full-body CT examinations, as reported by Toqueton et al.^(4)^.

In 55.9% of the CT scans analyzed, iodinated contrast was used, which can be worrisome because of the risk of adverse reactions, such as flushing, urticaria, angioedema, severe hypotension, bronchospasm, and contrast-induced nephropathy^(5)^. Incidental findings suggestive of neoplasia, requiring subsequent complementary examinations for better investigation, were seen in only 4.5% of the CT scans.

Extracranial soft tissue edema was seen on 11.9% of the head CT scans in our sample, all in cases categorized as traumatic emergencies. That was also the most prevalent finding in the study conducted by Lara Filho et al.^(6)^, although those authors did not state which cases were traumatic emergencies and which were not. In contrast, 59.3% of the head CT scans in our study were free of significant findings, a proportion considerably higher than the 23.5% reported by Lara Filho et al.^(6)^. That difference suggests that examinations in the emergency department are being ordered less judiciously than would be ideal.

Among the chest CT scans in our sample, the most common findings were pneumonia (in 23.7%), pleural effusion (in 12.9%), pulmonary contusion (in 5.4%) and bone fracture (in 4.3%). As previously stated, 26.5% of the chest CT scans were in cases of traumatic emergency. These data can be compared with those presented in the Seidel et al. study^(3)^, the only reference found that also demonstrated a difference between traumatic and non-traumatic findings, in which 26% of the chest CT scans revealed fracture and pulmonary contusion, with 17% of all chest CT scans having been indicated in cases of trauma.

Of the abdominal CT scans analyzed in the present study, only 6.3% were in cases of traumatic emergency. Among the non-traumatic emergencies, the most prevalent findings were obstructive uropathy, in 12.2%, enterocolitis, in 5.8%, and diverticulitis, in 5%, values consistent with those reported by Silva et al.^(7)^. However, we noted that those authors did not report the incidences of pyelonephritis and cholecystitis, which were reported in our study, revealing the particularities among the CT findings in non-traumatic cases of acute abdomen.

## CONCLUSION

In this study, we conducted an epidemiological analysis of patients who presented to the emergency department of a tertiary care hospital, as well as analyzing the type of demand for and most prevalent radiological findings on CT scans, data that are essential for good clinical practice at this level of care. The role of CT scans in the diagnosis and therapeutic management of patients with acute conditions became clear, because there was a significant prevalence of pathological findings in CT scans ordered by emergency department physicians. The importance of this study is that, given the scarcity of references on the subject in Brazil, radiological studies like this one contribute to improving the quality of patient care, forming a solid basis for clinical decisions and increasing efficiency in the allocation of hospital resources.
